# The Multitarget Antinociceptive Compound Affinin and Its Effects on Hypothermia, Hypolocomotion, and Sickness Behavior in Lipopolysaccharide-Treated Mice

**DOI:** 10.3390/molecules30122554

**Published:** 2025-06-11

**Authors:** Beatriz A. Luz-Martínez, Juan M. Viveros-Paredes, Alejandra Rojas-Molina, César Ibarra-Alvarado

**Affiliations:** 1 Posgrado en Ciencias Químico Biológicas, Facultad de Química, Universidad Autónoma de Querétaro, Cerro de Las Campanas S/N, Querétaro 76010, Mexico; bluz116@alumnos.uaq.mx; 2 Laboratorio de Investigación y Desarrollo Farmacéutico, Departamento de Farmacobiología, Centro Universitario de Ciencias Exactas e Ingenierías, Universidad de Guadalajara, Guadalajara 44430, Mexico; juan.viveros@academicos.udg.mx; 3 Laboratorio de Investigación Química y Farmacológica de Productos Naturales, Facultad de Química, Centro Universitario, Universidad Autónoma de Querétaro, Querétaro 76010, Mexico; rojasa@uaq.mx

**Keywords:** affinin, *N*-alkylamide, analgesia, CB1, TRPV1, TRPA1, hypothermia, anti-inflammatory

## Abstract

Affinin (spilanthol) is the main bioactive alkylamide present in *Heliopsis longipes* roots, exerting antinociceptive and anti-inflammatory effects that involve the activation of TRP channels. Previous studies indicated that affinin reduces the LPS-induced increase in pro-inflammatory cytokine production in murine macrophages. However, no studies have evaluated whether affinin produces antinociceptive, anti-inflammatory, and behavioral effects in experimental animals treated with LPS, nor has the mechanism of action involved in these pharmacological effects been established. The present study evaluated whether affinin induces hypothermia, catalepsy, hypolocomotion, and analgesia and, moreover, whether the analgesia involves the activation of the CB1 cannabinoid receptor and TRPV1 and TRPA1 channels. Subsequently, the anti-inflammatory activity and behavioral effects induced by affinin (20 mg/kg) in mice were evaluated via LPS (2.5 mg/kg)-induced hypothermia. The results of the experiments indicate that the analgesic effect of affinin involves the activation of the CB1 cannabinoid receptors and the TRPV1 and TRPA1 channels. Additionally, affinin reduced the severity of LPS-induced hypothermia and attenuated the increase in TNF-α and IL-6 levels in serum. The results obtained demonstrate that affinin induces antinociceptive, anti-hypothermic, and anti-inflammatory activities, which involve the CB1 receptor and the TRPV1 and TRPA1 channels and the suppression of pro-inflammatory cytokines.

## 1. Introduction

A group of bioactive compounds with a wide structural diversity, *N*-alkylamides (alkamides) are found in more than 25 plant families and exert a variety of biological and pharmacological effects [1]. These metabolites have been found in high quantities in members of the Asteraceae family, such as *Spilanthes*, *Acmella*, and *Heliopsis* [2,3,4]. In Mexico, the roots of *Heliopsis longipes* (A. Gray) SF Blake (Asteraceae) were widely used by the Náhuatl civilization as a flavoring in food preparation [5]. Currently, its roots are used as a condiment in dishes, sauces, and beverages [4]. In traditional Mexican medicine, it is still used as an oral anesthetic, wherein chewing a piece of *Heliopsis longipes* root produces intense salivation and local anesthetic and analgesic effects [6].

Affinin (syn. Spilanthol), (2*E*, 6*Z*, 8*E*)-*N*-isobutyl-2,6,8-decatrienamide, is the main alkylamide present in *Heliopsis longipes* roots [4] and has potent analgesic and antinociceptive effects [7,8]. *N*-alkylamides are structurally similar to endocannabinoids, which induce analgesia when they bind to the cannabinoid type 1 receptor (CB1) [9]. Additionally, endocannabinoids are involved in the sensory function of pain, owing to the fact that they activate the transient receptor potential vanilloid 1 (TRPV1) and/or ankyrin 1 (TRPA1) channels [10,11]. Previous studies have shown that the antinociceptive effect of affinin involves the activation of TRPV1 [12]. Moreover, molecular docking assays show that affinin binds with high affinity to the CB1 receptor and the TRPA1 and TRPV1 channels, suggesting that affinin-induced pharmacological effects involve the activation of these molecular targets [12,13]. In order to evaluate the participation of cannabinoid receptors, CB1 agonists, such as phytocannabinoids (Δ^9^-THC, for example), are administered in murine models, inducing a series of behaviors known as the *cannabinoid tetrad*. These phenotypes are characterized by hypolocomotor activity, catalepsy, hypothermia, and analgesia [14].

Affinin regulates lipopolysaccharide (LPS)-induced inflammatory responses by inhibiting the production of IL-1β, IL-6, and TNF-α in RAW 264, a mouse macrophagic cell line, in part due to the inactivation of NF-κB [2]. These findings suggest that affinin may be a useful inhibitor of inflammatory mediators. Although inflammation is a protective response, its dysregulation can have harmful consequences, such as sepsis. Furthermore, the inflammatory response is often accompanied by pain. Endotoxins are potent inducers of pro-inflammatory immune responses. In particular, LPS is a classic endotoxin produced in the membrane of Gram-negative bacteria. It is also the best-known model due to its established role in inflammatory pathways [15].

The present study studied the effect of the acute administration of affinin on locomotor activity, body temperature, and analgesia, as well as the involvement of the CB1 receptor and the TRPV1 and TRPA1 channels in its antinociceptive effects. Furthermore, the anti-inflammatory activity and behavioral effects induced by affinin were also evaluated in mice with LPS-induced hypothermia.

## 2. Results

### 2.1. Effect of Affinin on Locomotor Activity

**Figure 1:**
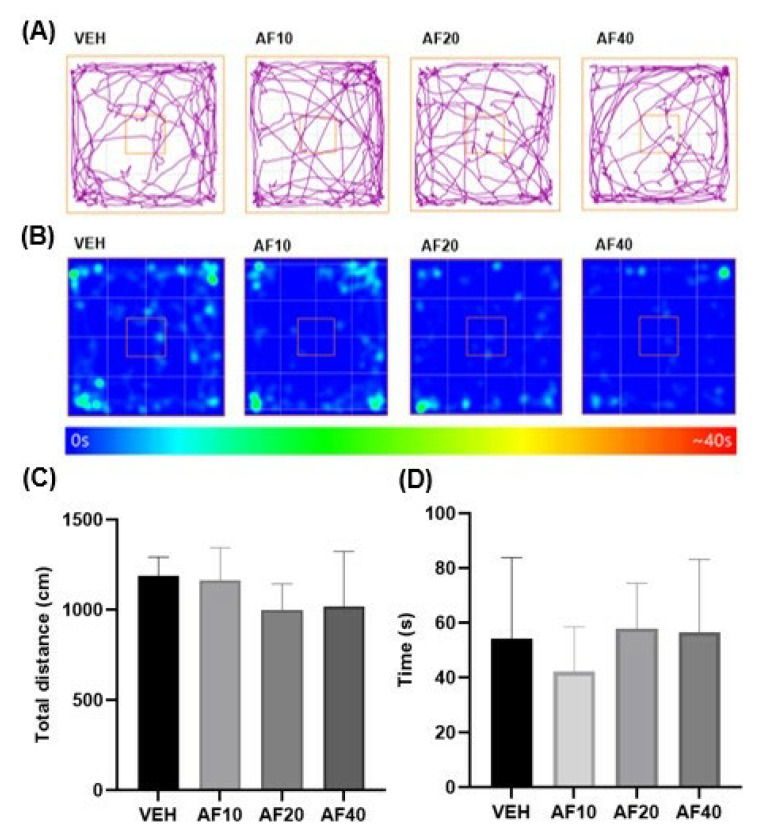
(**A**) Representative images of the trajectories (purple line) followed by the mice, the square indicates the center of the OFT; (**B**) heat maps of the effects of affinin on the OFT; (**C**) the total distance traveled for three minutes (cm); (**D**) total immobile time (s). Data were obtained from videos recorded and analyzed using the ANY-maze software. VEH—vehicle; AF—affinin, administered at 10, 20, and 40 mg/kg. Values are mean ± SD (*n* = 5); *p* > 0.05 using a one-way ANOVA followed by Tukey’s post hoc test.

Visualizations, using the ANY-maze software, of the data obtained from video recordings are shown in Figure 1A,B. The track plots (purple lines) show the animal’s trajectory during the test (Figure 1A), with the mice presenting exploratory behavior throughout the open field, crossing its center on several occasions, although they mainly walked along the walls. The behavior was similar in both the VEH group and the AF groups. The heat maps show where the animal spent most of the time (Figure 1B), with the mice observed to have remained longer near the walls and in the corners. Observation of the behavior of the mice in the OFT revealed that the affinin treatment (10, 20, and 40 mg/kg) did not affect the total distance traveled (Figure 1C; *F* [2,19] = 1164.9 ± 188.5, 995.5 ± 156.6 and 1018.1 ± 323.2 cm, respectively; *p* > 0.05), results that are similar to those observed for the vehicle (1185.7 ± 106.5 cm). The mobility of the mice was not observed to have been altered (Figure 1D; *F* [0,4383] = 54.1 ± 29, 42.1 ± 16, 57.9 ± 17, and 56.6 ± 26 s, respectively; *p* > 0.05) by the treatments applied either. These similarities can also be observed in the representative images of the trajectory followed by each mouse and the location where it spent most of the time during the test (Figure 1A,B, respectively).

### 2.2. Affinin-Induced Analgesia: The Cannabinoid Tetrad Test

**Figure 2:**
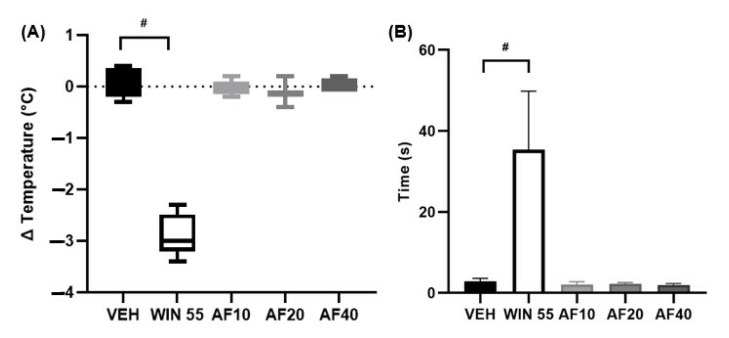
(**A**) Delta (Δ) of body temperature (°C); (**B**) catalepsy (s). VEH—vehicle; WIN 55—WIN 55,212-2; AF—affinin (at 10, 20, and 40 mg/kg). Values are mean ± SD (*n* = 5); ^#^ *p* < 0.0001 using a one-way ANOVA followed by Tukey’s post hoc test.

The cannabinoid tetrad test was performed to determine whether affinin induces the characteristic phenotypes of the CB1 receptor agonists, namely hypothermia, catalepsy, hypolocomotion, and analgesia. The tests were performed 2 h after the administration of affinin and WIN 55,212-2. The administration of affinin (10, 20, and 40 mg/kg) did not reduce the core body temperature (Figure 2A; Δ Temperature; *F* [210,0] = −0.04 ± 0.2, −0.13 ± 0.2 and 0.02 ± 0.1 °C; *p* > 0.05) of the mice tested, while the positive control, WIN 55, did induce hypothermia (*F* [210,0] = −2.87 °C ± 0.4; *p* < 0.0001). Affinin was not observed to induce catalepsy (10, 20, and 40 mg/kg; *F* [62,71] = 2.1 ± 0.7, 2.2 ± 0.4, and 1.9 ± 0.4 s, respectively) in the fixed-bar test (Figure 2B), unlike WIN 55,212-2, which induced marked catalepsy (*F* [62,71] = 35.9 ± 14.4 s; *p* > 0.0001).

**Figure 3:**
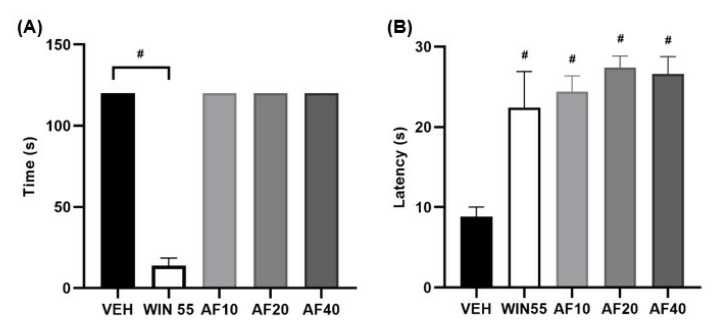
(**A**) Latency to falling in the rotarod test (s); (**B**) analgesia (s). VEH—vehicle; WIN 55—WIN 55,212-2; AF—affinin at 10, 20, and 40 mg/kg. Values are mean ± SD (*n* = 5); ^#^ *p* < 0.0001 vs. VEH using a one-way ANOVA followed by Tukey’s post hoc test.

The motor coordination of the mice was not observed to be affected by affinin (Figure 3A), with the animals remaining on the rotating bar for the 120 s for which they were trained to walk on it. The results obtained in the present study also showed that, in contrast to the vehicle group (VEH) (*F* [30,44] = 8.8 ± 1.3 s; *p* < 0.0001), affinin produced an analgesic effect in response to a thermal stimulus (10, 20, and 40 mg/kg; *F* [30,44] = 24.4 ± 2.1, 27.4 ± 1.5, and 26.6 ± 2.3 s, respectively) (Figure 3B), an effect observed for all doses administered.

**Figure 4:**
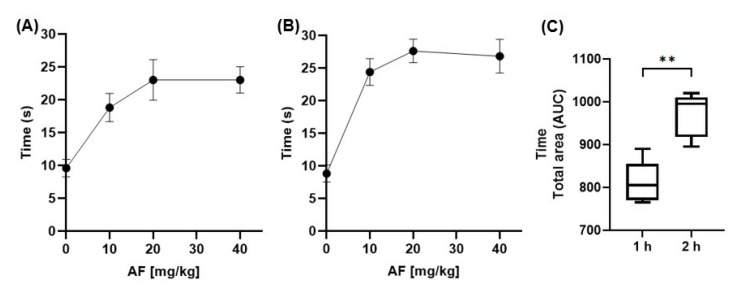
The analgesic effect of affinin. (**A**) One hour after the administration of affinin, (**B**) 2 h after the administration of affinin, and (**C**) the area under the curve (AUC). Values are expressed as the mean ± SD (*n* = 5); ** *p* < 0.001 using one-way ANOVA followed by Tukey’s post hoc test.

Affinin’s analgesic effect occurred 1 h after administration (Figure 4A; AF10 = 18.8 ± 2.2 s, AF20 = 23.0 ± 3.1 s and AF40 = 23.0 ± 2.0), and this effect was most pronounced 2 hours later (Figure 4C) (** *p* < 0.001). The effect was greater at doses of affinin (20 and 40 mg/kg) higher than 10 mg/kg. Therefore, the 20 mg/kg dose was selected to evaluate the molecular targets involved in its analgesic effect.

### 2.3. Involvement of the CB1 Receptors and the TRPA1 and TRPV1 Channels in the Analgesic Effect of Affinin

**Figure 5:**
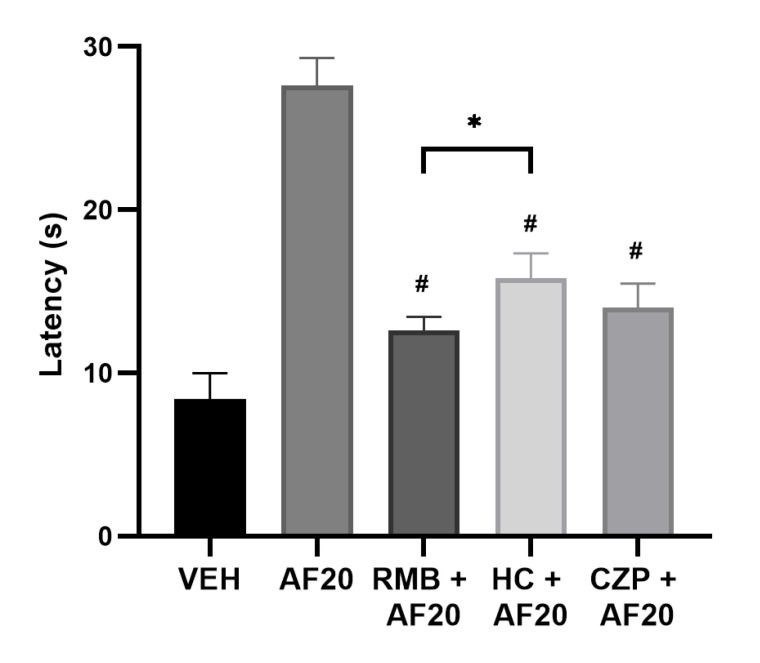
The effect of the prior administration of rimonabant (RMB), HC-030031 (HC), and capsazepine (CZP) on the analgesic effect of affinin (AF20) in the hot plate test. VEH—vehicle. Values are expressed as the mean ± SD (*n* = 5); ^#^ *p* < 0.0001 vs. AF20, * *p* < 0.01 using a one-way ANOVA followed by Tukey’s post hoc test.

The involvement of the CB1 receptors and TRPV1 and TRPA1 channels in affinin-induced analgesia was determined according to the protocol described in Figure 1B. The antagonism of the CB1 receptors and the TRPA1 and TRPV1 channels led to a decrease (Figure 5: *F* [107,1] = 12.6 ± 0.9, 15.8 ± 1.6, and 14.0 ± 1.3 s, respectively) in affinin-induced latency to thermal pain (27.6 ± 1.8 s). However, the analgesia was not found to be completely attenuated as it was for the VEH group (*p* < 0.01). The inhibition of the analgesic effect was lower when HC-030031 was administered.

### 2.4. Effects of Affinin on Behavior in LPS-Induced Hypothermic Mice

**Figure 6:**
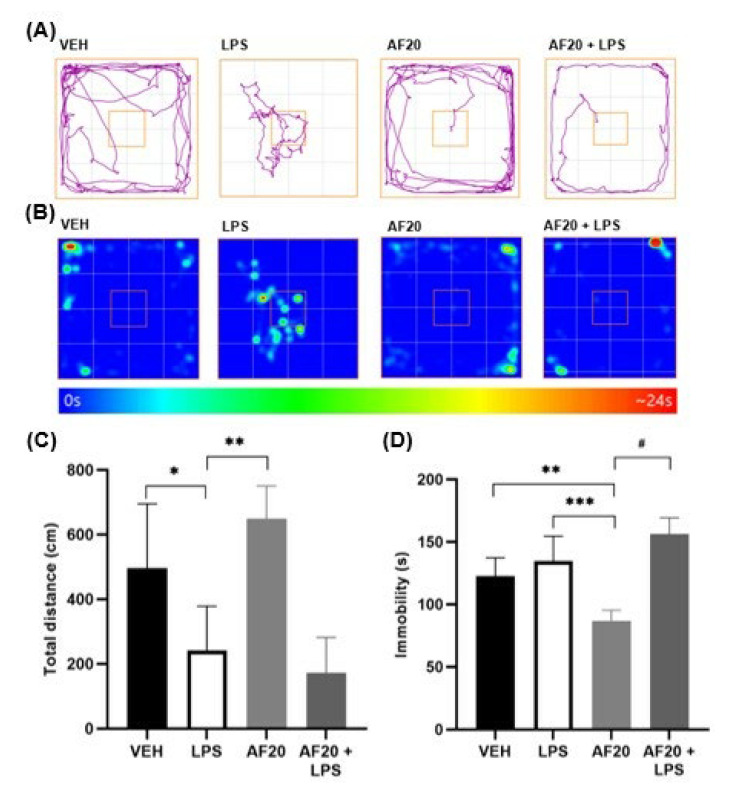
(**A**) Representative images of the trajectories (purple line) followed by the mice, the square indicates the center of the OFT; (**B**) heat maps of the effects of LPS and affinin on the OFT; (**C**) total distance traveled in the OFT (cm); (**D**) total immobile time (s). VEH—vehicle; LPS—lipopolysaccharide; AF20—affinin (20 mg/kg). Values are mean ± SD (*n* = 6); ^#^ *p* < 0.0001, *** *p* < 0.001, ** *p* < 0.01, * *p* < 0.05 using a one-way ANOVA followed by Tukey’s post hoc test. Data were obtained via video recordings and analyzed using the ANY-maze software.

Figure 6A,B show the track plot and heat map obtained using the ANY-maze software. The mice showed a higher preference for walking near the walls and in the corners of the open field and did not cross the center (Figure 6A). The heat maps indicate that the animals spent most of the time in the corners (Figure 6B). The locomotor activity of the hypothermic mice in the OFT and the results obtained for this element of the experiment indicated that the administration of LPS significantly reduced the distance traveled (Figure 6C; *F* [11,56] = VEH = 496.2 ± 198.8 vs. LPS = 241.6 ± 137.1 cm; *p* < 0.05). The total distance traveled by the VEH mice was significantly lower (Figure 6C) than that observed for the VEH group in the cannabinoid tetrad (Figure 1C). This may be associated with the measurement of rectal temperature, which was taken twice (basal and two hours later) in the cannabinoid tetrad, while, for LPS-induced hypothermia, measurements were made every half hour up to the four-hour mark, corresponding to ten measurements.

Mice administered AF20 traveled a greater, although not significantly, mean total distance (*F* [11,56] = 649.0 ± 101.7 cm) than those administered the vehicle. However, the administration of AF20 prior to LPS did not attenuate hypolocomotion (*p* > 0.05), while LPS administration was observed to slightly alter the immobile time (Figure 6D; *F* [19,97] = VEH = 122.5 ± 14.8 vs. LPS = 134.7 ± 20.0 s; *p* > 0.05), which, interestingly, significantly decreased for mice administered affinin (86.8 ± 8.5 s; *p* < 0.05) four hours after administration. However, the administration of affinin prior to LPS did produce a decrease in mobility (*F* [19,97] = 156.5 ± 12.8 s; *p* < 0.01). Figure 6B shows the heat maps indicating where the animals spent most of the time in the maze.

### 2.5. Evaluation of the Anti-Inflammatory Effect of Affinin in an LPS-Induced Hypothermia Model

**Figure 7:**
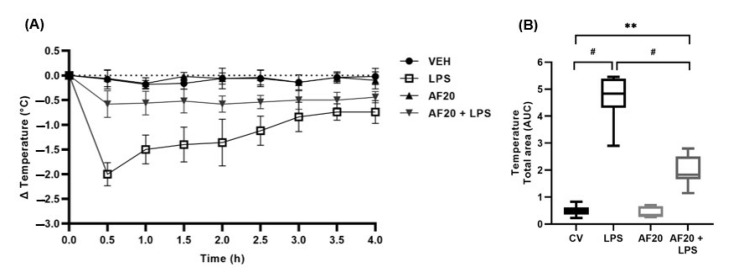
(**A**) Change in body temperature over time (h); (**B**) area under the curve (AUC) showing the body temperature change over four hours. VEH—vehicle; LPS—lipopolysaccharide; AF20—affinin at 20 mg/kg. Data show mean ± SD (*n* = 5); ^#^ *p* < 0.0001; ** *p* < 0.001 using a one-way ANOVA followed by Tukey’s post hoc analysis.

The present study evaluated whether the administration of affinin treatment to hypothermic mice showed protective effects. The application of an endotoxin challenge via LPS induced a decrease in temperature (Figure 7A), with a marked trough (−2 °C) observed 30 min after administration. While the level of hypothermia did fall over time, the animal had not completely recovered after four hours. It was observed that the pre-administration of affinin (20 mg/kg; 30 min) reduced the severity of LPS-induced hypothermia (Figure 7B; *F* [50,40] = 2.0 ± 0.6 vs. 4.7 ± 1.0 AUC; *p* < 0.0001).

### 2.6. Quantification of Serum Cytokines: IL-1β, TNF-α, and IL-6

**Figure 8:**
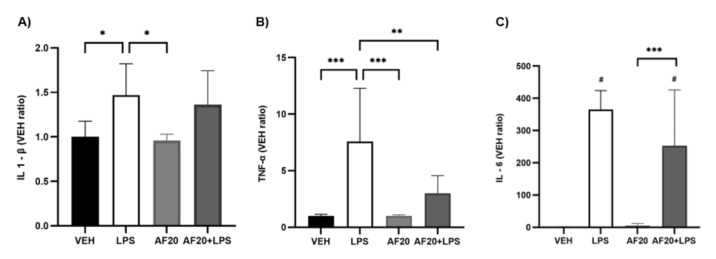
Levels of pro-inflammatory cytokines in the serum of BALB/c mice. (**A**) IL-1β; (**B**) TNF-α; (**C**) IL-6. VEH—vehicle; AF20—affinin (20 mg/kg); LPS—lipopolysaccharide. The values are expressed as the ratio of the mean VEH + SD (VEH = 1) (*n* = 6), ^#^ *p* < 0.0001 vs. VEH, *** *p* < 0.001, ** *p* < 0.01, * *p* < 0.05 using a one-way ANOVA followed by Tukey’s post hoc test.

One of the main characteristics of the administration of high doses of LPS is an exacerbated inflammatory response characterized by an increase in cytokine levels. The present study found that mice administered LPS (2 mg/kg) presented a higher level of IL-1β than the VEH group (Figure 8A; *F* [5,706] = 0.5-fold; *p* < 0.05) and an elevated production of both TNF-α (Figure 8B; *F* [17,71] = 6.6 times higher than the VEH group; *p* < 0.001) and IL-6 (Figure 8C; *F* [28,17] = 364.4 times higher than the VEH group; *p* < 0.0001). The administration of affinin alone did not induce an inflammatory response (*p* > 0.05), while prior treatment with AF20 attenuated increases in both TNF-α (*F* [17,71] = 3.0 ± 1.7 vs. 7.6 ± 5.1; *p* < 0.01) and IL-6 (*F* [28,17] = 252.8 ± 183.9 vs. 365.4 ± 63.2).

## 3. Discussion

*H. longipes* has been traditionally used in Indigenous herbalism in Mexico, where it is known as *chilcuague*. Currently, this plant species is sold at medicinal herb stalls throughout most of the country [6]. Its root is used as a condiment in sauces and spicy foods because it has a flavor similar to chili [7]; in addition, the roots of *H. longipes* are used as both an analgesic and local anesthetic. People chewing the roots of this plant experience an intense sensation of numbness and tingling in the lips, tongue, and mouth, further to the stimulation of salivation. Affinin is the main bioactive component of the roots of this plant species [6].

The present study evaluated, by means of the OFT, the behavior of mice under the influence of different doses of affinin (10, 20, and 40 mg/kg, p.o.) (Figure 1). The results of the experiments indicated that the administration of affinin did not exert any change in locomotor activity, namely, the total ambulatory distance covered by the animal. The results obtained coincide with a previous study, in which the administration of affinin via a different route (1–30 mg/kg, i.p.) did not induce significant changes in ambulatory activity at any of the doses tested [16].

The analgesic and antinociceptive effects of affinin have been evaluated in several experimental murine pain models [7,8,17,18]. These pharmacological effects are associated with an increase in the release of gamma-aminobutyric acid (GABA) in the mouse brain [17], resulting in the activation of the opioidergic and serotonergic systems, as well as the NO/cGMP pathway [8] and the TRPV1 channels [12]. Our research group has previously demonstrated that affinin induces the concentration-dependent relaxation of the rat aorta [4] while also observing the mediation of affinin-induced vasodilation via the activation of the NO/cGMP pathway, CB1 receptors, and TRPV1 and TRPA1 channels. Furthermore, molecular docking assays have indicated that affinin binds to these molecular targets [13]. Because CB1 receptor agonists can act on the central nervous system (CNS), the cannabinoid tetrad test was used to determine whether affinin, as mediated by cannabinoid receptors, induces the following effects on rodent behavior: hypothermia, catalepsy, hypolocomotion, and analgesia [19]. While the administration of affinin did not modify body temperature, responsiveness, or locomotor activity, in a similar manner to the cannabinoid agonist WIN 55,212, it did increase the latency to pain induced by a thermal stimulus (Figure 3B). The exposure of peripheral sensory nerve endings to elevated temperatures evokes sensations of heat or pain, with the sensation of pain generally occurring at temperatures of 42 to 48 °C [20]. The present study used the hot plate test to induce pain in BALB/c mice via a thermal stimulus (54–55 °C), generating a steep temperature gradient associated with the activation of Aδ fibers. Small Aδ fibers are myelinated and present faster conduction velocities (4–36 m/s) than C fibers, which are unmyelinated [21]. These findings suggest that affinin could be used as a potent analgesic to treat disorders affecting small myelinated Aδ fibers, such as small fiber neuropathy, without the psychotropic effects produced by some cannabinoid agonists, such as Δ^9^ THC.

*N*-alkylamides exert a wide range of pharmacological effects through various mechanisms of action [22]. For example, guineensine is an endocannabinoid uptake inhibitor with anti-inflammatory and analgesic effects [23]. In addition, capsaicin is an agonist of the TRPV1 channel and has vasodilatory, antinociceptive, anti-cancer, and antimicrobial properties [24]. Furthermore, affinin has shown both analgesic and antinociceptive activity, which may involve the activation of the opioidergic, serotonergic, and GABAergic systems, as well as the TRPV1 channels [7,8,12].

Taking into account the foregoing findings, the present study evaluated whether the analgesic effect of affinin occurred due to the participation of the CB1 receptors and the TRPV1 and TRPA1 channels. The analgesic effect induced by affinin was inhibited when the CB1 receptors were antagonized (Figure 5). The results of the present study coincide with our previous study, which demonstrated, via ex vivo and in silico experiments, that affinin can interact with these receptors [13]. The abolition of pain behavior is attributed to the suppression of nociceptive transmission. The activation of the CB1 receptor inhibits the release of neurotransmitters, including glutamate, GABA, glycine, acetylcholine, norepinephrine, dopamine, and serotonin [25]. Cannabinoid-induced antinociception results from the disinhibition of GABAergic output neurons in the periaqueductal gray, leading to the activation of descending inhibitory pain pathways [26].

The TRPV1 and TRPA1 channels are members of the structurally related superfamily of nonselective cation channels, which are highly permeable to calcium [27]. de La Rosa-Lugo et al. (2017) [12] showed both that affinin administration in the orofacial region produced antinociception in female mice and that this effect was inhibited by capsazepine, thus suggesting the involvement of TRPV1 channels. The present study found that the inhibition of the TRPV1 channel abolished the antinociceptive effect of affinin (Figure 5). The TRPA1 channel is widely expressed in neuronal (e.g., the dorsal root and sensory neurons of the trigeminal ganglion) [28] and non-neuronal (e.g., arterial endothelial cells and cardiomyocytes) cells [29,30]. This channel is activated by various chemical compounds, including allyl isothiocyanate (AITC), cinnamaldehyde [31], allicin [32], capsaicin, and affinin [12,33]. It has been reported that the activation of the TRPA1 channel in the spinal cord by the cannabinoid ∆^9^-tetrahydrocannabiorcol reduced the occurrence of nociceptive behavior in mice [34]. The results of the present study suggest that the TRPA1 channel also participates in the analgesic effect of affinin but to a lesser degree than either the CB1 receptor or the TRPV1 channel. These findings seem to coincide with our previous results, where docking analysis conducted on the interaction of affinin with these receptors showed that this *N*-alkylamide binds with greater affinity to CB1, followed by TRPV1 and then TRPA1 (docking score (kcal/mol): −8.57, −7.21, and −6.18, respectively) [13]. Therefore, it can be proposed that the analgesic effect of affinin comprises several pathways, including the CB1 receptors and the TRPV1 and TRPA1 channels.

The TRPA1 and TRPV1 channels are coexpressed in a subset of sensory nerves and non-neuronal cells, while a functional interaction between these receptors that regulates inflammatory processes has been identified [35]. The activation of the TRPV1 channel has been found to play an anti-inflammatory role by increasing intracellular Ca^2+^ and activating the PI3K/Akt signaling pathway, which, in turn, inhibits NF-κB activation [36]. Furthermore, activating the TRPA1 channel with cinnamaldehyde reduces the levels of IL-1β, TNF-α, and IL-6. Cinnamaldehyde inhibits intracellular ROS production and suppresses TLR4 and NOX4 during LPS-induced endotoxemia [37].

As it has been previously reported that injection with LPS is associated with sickness behavior in rodents [38], the present research sought to ascertain whether affinin had a protective effect on LPS-induced hypolocomotion. The results obtained showed that LPS decreased the level of locomotor activity in mice (Figure 6C). In mammals, high doses of LPS (2 mg/kg) significantly decrease locomotor activity [23]. Interestingly, the present study found that the administration of AF20 increased the distance traveled by the mice and decreased their immobile time (Figure 6C,D). These results are promising, as a requirement for analgesic drugs is that they do not affect cognitive functions. For example, paracetamol, a medication commonly used to alleviate pain and reduce fever, has been found to produce a dose-related (50 or 300 mg/kg) increase in anxiety-like behavior and a deterioration in cognitive performance in rodents [39].

It is important to note that, despite the selective usefulness of in silico or in vitro models in the drug development process, none of the foregoing prototypes reproduce that which occurs in a complete organism. Therefore, the present study evaluated, in an in vivo model, whether affinin has the potential to inhibit the systemic inflammatory process. Endotoxemic shock produced by high doses of LPS is characterized by an increase in the release of pro-inflammatory cytokines, known as a *cytokine storm*, and a decrease in body temperature [40]. As hypothermia correlates with poor prognosis in both animal models and humans, the present study analyzed the thermoregulatory response to affinin in animals injected with a high dose (2.5 mg/kg) of LPS. In the present study, AF20 inhibited the decrease in Δ temperature (°C) otherwise observed in mice with LPS-induced hypothermia (Figure 7B), while the high levels of pro-inflammatory mediators, including TNF-α and IL-6, decreased significantly (Figure 8B,C). Differences in cytokine kinetics may be caused by different mechanisms of secretion and function. IL-1β and TNF-α play a role as pro-inflammatory cytokines, with the release of IL-1β increasing at 2 h of exposure to LPS, while TNF-α release increases after 4 h [41]. It has been reported that IL-6 plays a consecutive dual role as both a pro-inflammatory and anti-inflammatory cytokine, which is why its levels were extremely high after 4 h of exposure, at which point the inflammation was attenuating and the level of anti-inflammatory molecules was increasing [42].

Previous studies have shown that, in in vitro models, affinin regulates the secretion of pro-inflammatory cytokines. A marked inhibitory effect on the production of pro-inflammatory mediators was observed in RAW 264.7 murine macrophages stimulated with LPS and then exposed to affinin [2]. This effect was attributed, in part, to a decrease in the activation of nuclear factor kappa β (NF-κβ). It has also been reported that affinin exerts a significant inhibitory effect on the release of IL-8 and TNF-α in neutrophils isolated from human blood and activated via stimulation with LPS [43]. This finding suggests that affinin may regulate body temperature by reducing pro-inflammatory cytokines, TNF-α, and, at a lower level, IL-6. The present study shows, for the first time, the anti-hypothermic effect of affinin. Surprisingly, affinin was observed to inhibit LPS-mediated hypothermia and exacerbate pro-inflammatory cytokine production, but not hypolocomotion. This could be due to elevated levels of IL-1β. It is well known that IL-1β is an important cytokine for the induction of sickness behavior [44]. However, IL-1B is not the sole cytokine that mediates sickness behavior, with IL-6, for example, potentiating the depressive behavioral effects of IL-1β [45].

**Figure 9:**
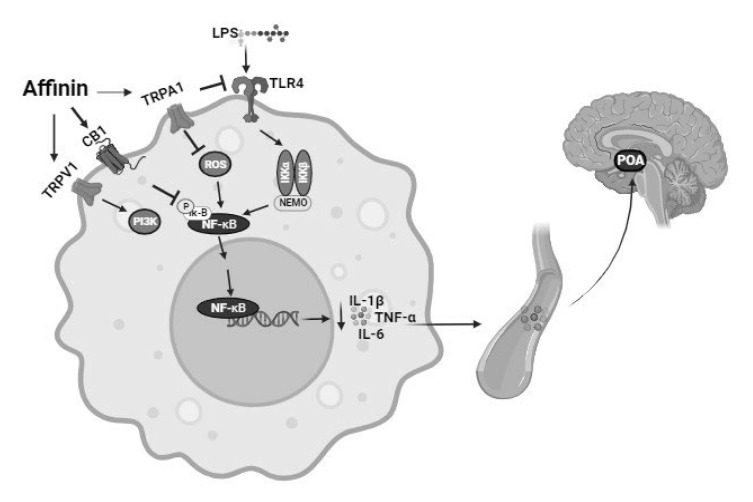
The pathway involved in the anti-inflammatory effect of affinin. Affinin attenuates hypothermia caused by LPS by preventing the exacerbated production of pro-inflammatory cytokines. CB1—cannabinoid type 1 receptor; POA—hypothalamic preoptic area; IKK—IkB-kinase; IkB—inhibitor of kappa B; IL-1β—interleukin-1β; IL-6—interleukin-6; LPS—lipopolysaccharide; NEMO—NF-κB essential modulator; NF-kB—nuclear factor kappa-light-chain-enhancer B cells; PI3K—phosphatidylinositol-3-kinase; ROS—reactive oxygen species; TLR4—toll-like receptor 4; TRPA1—transient receptor potential ankyrin 1 channel; TRPV1—transient receptor potential vanilloid 1 channel; TNF-α—tumor necrosis factor-α.

Lipopolysaccharide is recognized by the innate immune system through toll-like receptors (TLRs) found on many types of immune cells, such as monocytes and macrophages. The binding of LPS to its native receptor, TLR-4, activates an intracellular cascade that ultimately causes the translocation of NF-κB to the nucleus, where it initiates the transcription of inflammatory cytokines, such as IL-1β, TNF-α, and IL-6 [40]. Activated macrophages secrete cytokines into circulation, especially TNF-α and IL-6, which, once circulating, can send signals to the CNS through cytokine receptors present in either the blood–brain barrier or the vagus nerve. This signaling may be responsible for hypothalamic activation, which can induce the hypothermic response, given that it is known that the hypothalamic preoptic area (POA) is the main integrative site of the brain for thermoregulation [46]. Therefore, affinin could attenuate the hypothermia caused by LPS by preventing the exacerbated production of pro-inflammatory cytokines, such as TNF-α and IL-6. Figure 9 shows the signaling pathway the present study posits as being involved in the anti-inflammatory effect of affinin.

The anti-inflammatory properties of affinin may be beneficial for the treatment of SARS-CoV-2 infection, which induces an excessive and prolonged cytokine/chemokine response (e.g., TNF, IL-6, IL-1β, IFN-γ, MCP-1). One consequence of this cytokine storm is the induction of apoptosis in pulmonary epithelial and endothelial cells, eventually leading to hypoxia. Timely control of the cytokine storm in its early stages—by using immunomodulators and cytokine antagonists, as well as by reducing pulmonary inflammatory cell infiltration—has been shown to be critical for improving treatment outcomes and reducing mortality in patients with COVID-19 [47].

Considering our previous data demonstrating the analgesic and anti-inflammatory effects of affinin, a more in-depth characterization of the key pathways underlying these effects is still needed. Future research would focus on a chronic inflammatory pain model, such as the induction of monoarthritis via intra-articular injection of CFA in mice. In addition, we evaluated CB1 receptor expression and the levels of its endogenous agonists—2-arachidonoylglycerol (2-AG) and N-arachidonoylethanolamine (AEA)—in the somatosensory cortex and brain. Notably, these endocannabinoids also exert pharmacological effects on TRPV1 channels [48].

In summary, the findings of the present study demonstrate that affinin presents favorable antinociceptive, anti-hypothermic, and anti-inflammatory activities, which are related to the involvement of the CB1 receptors and the TRPV1 and TRPA1 channels, as well as the suppression of pro-inflammatory cytokines. These findings validate the use of affinin in the treatment of the pain and hypothermia associated with various conditions. To date, affinin has demonstrated promising efficacy in in vivo models of nociceptive and inflammatory pain. However, further research is needed to confirm its analgesic effects in models of neuropathic and chronic pain. Future studies should focus on evaluating the potential of affinin for treating chronic pain associated with small nerve fiber dysfunction. In addition, alternative assays should be employed to assess pain levels, such as the von Frey test, which is commonly used to evaluate mechanical allodynia in rodents.

## 4. Materials and Methods

### 4.1. Animals and Ethical Statement

All the experiments were conducted on male BALB/c mice (weighing 25 ± 2 g), which were kept at a temperature of 25 ± 2 °C on a 12 h light/dark cycle, with water and food provided ad libitum. The mice were provided with physical environmental enrichment, including cardboard tubes and paper for nest building. The animals were supplied by the Centro de Investigación Biomédica de Occidente-Instituto Mexicano del Seguro Social. All animal handling and experimentation was carried out in accordance with both the Official Mexican Standard NOM-062-ZOO-1999 [49] and the standard established by the International Council for Laboratory Animal Science. The animal cages were properly labeled, and the subject number was marked on the mouse’s tail with a different color for each group. The protocol applied in the present study was approved by the Institutional Committee for Care and Use of Laboratory Animals of the University of Guadalajara, under protocol code CUCEI/CINV/CICUAL-04/2022.

### 4.2. Extraction and Isolation

Dried *Heliopsis longipes* (Asteraceae) roots were collected in Peñamiller, Querétaro, Qro., Mexico (DMS: 21°03′01.9″ N 99°49′55.1″ W). The voucher specimens (*H. longipes* vouchers J.E. Castro R.1. and R.2.) were deposited in the Jerzy Rzedowski herbarium (QMEX). The roots were dried and ground and then macerated with dichloromethane at a ratio of 1:10 (*m/v*), with the solvent then removed using a rotary evaporator (Heidolph^®^ VV 2000, Schönwalde-Glien, Germany) to obtain the dry extract. One hundred grams of the dichloromethane extract was fractionated by normal-phase column chromatography, with the affinin then isolated as previously described [4]. The purity of affinin was determined by HPLC-PDA, using an HPLC chromatograph (Waters 600 Associates, Milford, MA, USA) with a photodiode array detector (Waters 2998). The purity of the isolated affinin was >94%, while a dry-weight yield of 19 g/kg root was obtained.

### 4.3. Drugs

LPS (*Escherichia coli*, serotype O111:B4), WIN 55,212-2, rimonabant, capsazepine, and sodium carboxymethyl cellulose were purchased from Sigma-Aldrich (St. Louis, MO, USA). HC-030031 was purchased from Cayman Chemical (Ann Arbor, MI, USA). WIN 55,212-2, rimonabant, HC-030031, and capsazepine were dissolved in dimethyl sulfoxide (DMSO), while LPS was suspended in a 0.9% saline solution.

### 4.4. Open-Field Test

**Figure 10:**
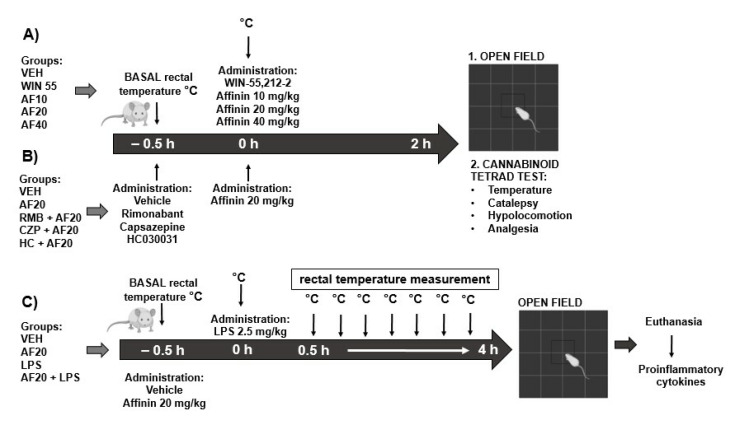
A diagram of individual experiments: (**A**) OFT; (**B**) cannabinoid tetrad test; (**C**) LPS-induced hypothermia. The groups are labeled as follows: 1. VEH—vehicle; 2. WIN 55—WIN 55,212-2; 3. AF10—affinin (10 mg/kg); 4. AF20—affinin (20 mg/kg); 5. AF40—affinin (40 mg/kg); 6. Antagonist + AF20; 7. LPS—lipopolysaccharide.

The behavior of the mice was observed both under the influence of different doses of affinin (AF: 10, 20, 40 mg/kg, p.o.) (Figure 10A) and in conditions of LPS-induced hypothermia (2.5 mg/kg, i.p.) (Figure 1C). The open-field test (OFT) assessed spontaneous locomotor activity using a white polyvinyl chloride (PVC) box (40 × 40 × 40 cm) with a black background. Each mouse was placed in the center of the open field and allowed to explore freely for three minutes, with the experimenter out of sight. The behavior exhibited by the mice was recorded using a video camera. The total distance traveled (cm) by the mice and their immobility (s) were measured using the ANY-maze software, version 7.1 (Stoelting Co., Wheat Lane, IL, USA). Figure 10 shows the experimental procedure that was followed for each of the groups. Excel templates were used to indicate the treatment and the exact time of the measurements.

### 4.5. Cannabinoid Tetrad Test

WIN 55,212-2 was used as a positive control to demonstrate the behavior induced by the CB1 agonist. The cannabinoid tetrad test evaluates four biological effects that were first described for the CB1 agonist Δ^9^- tetrahydrocannabinol (THC), which induces hypothermia (low body temperature), catalepsy (prolonged muscular rigidity and immobility), hypolocomotion (low locomotor activity), and analgesia (decreased sensitivity to pain). The animals were randomly assigned to the experimental groups (*n* = 6) and administered the following, depending on the group assigned: WIN 55,212-2 (WIN 55: 3 mg/kg, i.p.); affinin (AF: 10, 20, 40 mg/kg, p.o.); or vehicle, namely, sodium carboxymethyl cellulose 1% (VEH). In the present study, the tetrad test was performed two hours after the administration of the drugs (Figure 1B).

To evaluate hypothermia, the rectal temperature was recorded using a microprobe thermometer (BAT-12; Physitemp Instruments, LLC, Clifton, NJ, USA). To obtain rectal temperature, the mouse was hand-restrained and placed on a horizontal surface (cage lid) with the tail lifted, after which a probe was gently inserted into the rectum to a constant depth of 1 cm and removed after each reading. The basal body temperature was recorded both before the administration of either drug and then two hours after administration, with the change in body temperature expressed as the difference between the temperatures obtained (Δ °C).

Catalepsy was measured using a fixed bar placed 4 cm above the testing platform onto which the animal’s front paws were placed while its hind paws were placed on the floor. Latency in this position was measured until the mouse initiated movement, descended from the bar, or had remained in that position for over 120 s.

Hypolocomotion was evaluated by measuring learned locomotor activity using the rotarod test (Erweka; Heusenstamm, Germany), in which the animals were trained to walk on a rotating bar at 4 rpm for 2 min, with the time (s) for which the animals remained without falling from the bar then recorded. The mice were trained for three consecutive days until they were able to remain walking on the rotating bar for 120 s.

The hot plate test was used to assess analgesia in the form of thermal pain sensitivity. The animals were placed on a hot plate, at a temperature of 55 ± 1 °C (Thermo Scientific; Waltham, MA, USA), located inside a Plexiglas cylinder, with the latency (s) to the first nociceptive response (such as jumping, licking a hind paw, or the flinching of one of the paws) then measured. When the animal showed no signs of pain after 30 s, it was removed from the plate to prevent tissue damage. Analgesia was evaluated using blinded tests conducted by an experimenter different from the one who performed the group assignments.

To determine the involvement of the CB1 receptors and the TRPV1 and TRPA1 channels, a pretreatment with rimonabant (CB1 antagonist; 3 mg/kg, i.p.), capsazepine (TRPV1 antagonist; 5 mg/kg, i.p.), or HC-030031 (TRPA1 antagonist; 10 mg/kg, i.p) was administered 30 min before affinin (20 mg/kg, p.o.) or vehicle administration, with the hot plate test then conducted 2 h later (Figure 10B). The doses were set based on reports in the literature and the in-house experience of the research group working with the test compounds used [48,50,51].

### 4.6. LPS-Induced Hypothermia

The present study evaluated whether the affinin treatment administered to endotoxemic mice showed protective effects. The VEH and AF20 + LPS groups were orally administered the vehicle or affinin (20 mg/kg), respectively, 30 min before the intraperitoneal injection of 0.9% saline solution or LPS (2.5 mg/kg) (see Figure 10C). When high doses of LPS (2.5 mg/kg, i.p.) are administered in mice, an inflammatory reaction occurs, with cytokine release and hypothermia occurring. The basal temperatures were measured before the administration of any of the treatments, while rectal temperatures were determined every 30 min after LPS injection for 4 h and expressed as the mean and standard deviation of the basal temperature (Δ °C). The effect of affinin on LPS-induced hypothermia was evaluated based on the area under the Δ temperature–time curve after dosing (AUC_0–4h_). The anti-hypothermic effect of affinin was calculated as Δ AUC_0–4h_. Videos of the application of the OFT were recorded 4 h after LPS injection, according to the protocol described in Figure 1C. No mortality was associated with LPS treatment at the administered dose. At the end of the experiment, all the mice were sacrificed, and blood samples were obtained for the measurement of inflammatory cytokines.

### 4.7. Cytokine Measurements

Inflammatory cytokine levels were determined via blood serum. The quantification of IL-1β, TNF-α, and IL-6 was performed using the MILLIPLEX^®^ Mouse Cytokine/Chemokine Magnetic Bead Panel Kit (Cat #: MCYTOMAG-70K. Merck KGaA; Darmstadt, Germany), in accordance with the manufacturer’s instructions.

### 4.8. Statistical Analysis

Simple randomization was employed to assign subjects to treatment groups. The OFT video recordings were analyzed using the ANY-maze behavioral tracking system (7.1; Stoelting Co., Wheat Lane, IL, USA) [52]. The Kolmogorov–Smirnov and Shapiro–Wilk tests were performed to determine the normality of the data, with results expressed as the mean ± SD. A one-way analysis of variance (ANOVA) followed by Tukey’s test was used to compare differences between treatments. A confidence level of *p* < 0.05 was considered statistically significant. The analysis was carried out using the GraphPad Prism software, version (9.2.0; IBM, Inc., Chicago, IL, USA).

## Data Availability

The original contributions presented in this study are included in the article. Further inquiries can be directed to the corresponding author.
